# Arterial Spin Labeling Reveals Disrupted Brain Networks and Functional Connectivity in Drug-Resistant Temporal Epilepsy

**DOI:** 10.3389/fninf.2018.00101

**Published:** 2019-03-06

**Authors:** Ilaria Boscolo Galazzo, Silvia Francesca Storti, Anna Barnes, Bianca De Blasi, Enrico De Vita, Matthias Koepp, John Sidney Duncan, Ashley Groves, Francesca Benedetta Pizzini, Gloria Menegaz, Francesco Fraioli

**Affiliations:** ^1^Department of Computer Science, University of Verona, Verona, Italy; ^2^Institute of Nuclear Medicine, University College London, London, United Kingdom; ^3^Department of Medical Physics, University College London, London, United Kingdom; ^4^Department of Biomedical Engineering, School of Biomedical Engineering & Imaging Sciences, King’s Health Partners, King’s College London, London, United Kingdom; ^5^Department of Clinical and Experimental Epilepsy, Institute of Neurology, University College London, London, United Kingdom; ^6^Department of Neuroradiology, University Hospital Verona, Verona, Italy

**Keywords:** arterial spin labeling, perfusion, functional connectivity, resting-state, ICA, epilepsy

## Abstract

Resting-state networks (RSNs) and functional connectivity (FC) have been increasingly exploited for mapping brain activity and identifying abnormalities in pathologies, including epilepsy. The majority of studies currently available are based on blood-oxygenation-level-dependent (BOLD) contrast in combination with either independent component analysis (ICA) or pairwise region of interest (ROI) correlations. Despite its success, this approach has several shortcomings as BOLD is only an indirect and non-quantitative measure of brain activity. Conversely, promising results have recently been achieved by arterial spin labeling (ASL) MRI, primarily developed to quantify brain perfusion. However, the wide application of ASL-based FC has been hampered by its complexity and relatively low robustness to noise, leaving several aspects of this approach still largely unexplored. In this study, we firstly aimed at evaluating the effect of noise reduction on spatio-temporal ASL analyses and quantifying the impact of two ad-hoc processing pipelines (basic and advanced) on connectivity measures. Once the optimal strategy had been defined, we investigated the applicability of ASL for connectivity mapping in patients with drug-resistant temporal epilepsy vs. controls (10 per group), aiming at revealing between-group voxel-wise differences in each RSN and ROI-wise FC changes. We first found ASL was able to identify the main network (DMN) along with all the others generally detected with BOLD but never previously reported from ASL. For all RSNs, ICA-based denoising (advanced pipeline) allowed to increase their similarity with the corresponding BOLD template. ASL-based RSNs were visibly consistent with literature findings; however, group differences could be identified in the structure of some networks. Indeed, statistics revealed areas of significant FC decrease in patients within different RSNs, such as DMN and cerebellum (CER), while significant increases were found in some cases, such as the visual networks. Finally, the ROI-based analyses identified several inter-hemispheric dysfunctional links (controls > patients) mainly between areas belonging to the DMN, right-left thalamus and right-left temporal lobe. Conversely, fewer connections, predominantly intra-hemispheric, showed the opposite pattern (controls < patients). All these elements provide novel insights into the pathological modulations characterizing a “network disease” as epilepsy, shading light on the importance of perfusion-based approaches for identifying the disrupted areas and communications between brain regions.

## Introduction

The concept of the brain as a complex network characterized by structurally and functionally connected elements has gained a relevant place in the current research (Nunez, 2010). Understanding how brain regions specifically communicate and how information is integrated across networks remain one of the greatest challenges to deal with in the field of connectomics. In recent years, functional connectivity (FC) in resting-state has emerged as a valuable mean to characterize the intrinsic brain architecture (Biswal et al., 2010). Indeed, since the seminal work by Biswal et al. (1995) which proved the existence of synchronized spontaneous activity (Biswal et al., 1995), FC has become an increasingly popular tool to study the correlated activity in the absence of an explicit task and to identify functionally connected brain networks, generally known as resting-state networks (RSNs).

So far, the majority of FC studies has been based on resting-state fMRI (rs-fMRI) with the blood-oxygenation-level-dependent (BOLD) contrast thanks to its simplicity, high availability, good sensitivity, and temporal resolution (Van Dijk et al., 2010; Friston et al., 2014). BOLD signal fluctuations, however, represent only an indirect measure of neural activity, are concealed by several physiological and noise contributions, and often suffer from draining vein contamination, which limits the spatial specificity to the site of the neural activity. Moreover, these signals do not provide quantitative information as they result from the overall contributions of cerebral blood flow (CBF), cerebral blood volume (CBV) and cerebral metabolic rate of oxygen consumption (CMRO_2_), and therefore only variations between states can be derived (Chen et al., 2015). To overcome these limitations and achieve a more direct measure of brain metabolism, arterial spin labeling (ASL) has been increasingly adopted as a novel functional technique. This non-invasive MRI modality exploits magnetically labeled arterial blood water as an endogenous tracer for quantifying brain perfusion in physiological units (Detre et al., 1992). ASL acquisitions consist of label and control images, which are repeated over time to increase the signal-to-noise ratio (SNR) and whose subtraction generates a perfusion weighted map. ASL has been initially developed as an alternative to the more invasive techniques from nuclear medicine, among which the [^15^O]-H_2_O positron emission tomography (PET) is still considered the gold standard. Despite the limited ASL availability in clinical settings, several studies have demonstrated its good reliability and scan stability, as well as its ability to produce accurate and reproducible CBF measurements compared to water-PET (Alsop et al., 2015; Fan et al., 2016), suggesting its viability as robust imaging method.

In light of these promising findings and considering its closer coupling with the neural activity, ASL has started to be used for localizing the general linear model activations in task-based paradigms (Tjandra et al., 2005; Raoult et al., 2011; Boscolo Galazzo et al., 2014; Ghariq et al., 2014) and, in very limited studies, for resting-state FC analysis in healthy subjects (Viviani et al., 2011; Liang et al., 2013, 2014; Jann et al., 2013, 2015; Storti et al., 2017b, 2018). Biswal et al. (1997) were the first to demonstrate resting-state FC based on ASL, although restricting the analysis to a single slice, while De Luca et al. (2006) reported the possibility of detecting five of the most common RSNs using perfusion fluctuations (Biswal et al., 1997; De Luca et al., 2006). These ideas were taken forward by Chuang et al. (2008) who developed a framework for the computation of perfusion-based FC, by removing among the others the BOLD contaminations from ASL signals. That work suggested the need to properly clean the ASL data before all the FC analyses (Chuang et al., 2008). Indeed, as ASL suffers from low SNR, low temporal resolution and low sensitivity, it requires appropriate pre-processing to separate noise components from the true signal, thus increasing the sensitivity of ASL-based FC findings. While the effect of different cleaning pipelines on connectivity measures has been widely investigated in BOLD fMRI (Griffanti et al., 2015; Carone et al., 2017), this topic has only recently started to be addressed in ASL. In particular, Jann et al. (2016) investigated the effect of noise reduction on ASL-based connectivity analysis, focusing on nuisance regression approaches with the same variables used in BOLD and demonstrating that the connectivity results were highly affected by the choice of a pre-processing pipeline.

Besides choosing the optimal cleaning pipeline, dealing with the different connectivity methods currently available can be similarly difficult, especially with ASL data. There are two commonly used approaches for studying FC from rs-fMRI signals that are independent component analysis (ICA) and seed-based analysis. The first one is a data-driven method that allows separating a set of signals into spatially independent components (ICs) and associated time courses (McKeown et al., 1998). ICA-based studies have identified several ICs that correspond to functionally relevant networks as the visual and sensory-motor loops (Beckmann et al., 2005), while others purely reflect artefactual processes (e.g., head motion, physiological fluctuations, MRI hardware). Therefore, ICA is a successful technique not only to derive FC measures but also to clean the data, as once the noise ICs are identified they can then be regressed out from the signals. Of note, several authors have started to use and recommend ICA-based artifact removal for BOLD fMRI (Griffanti et al., 2014; Pruim et al., 2015), but to the best of our knowledge no previous studies have investigated the impact of ICA-based denoising on ASL data and corresponding FC measures. The alternative approach, seed-based analysis, relies on prior hypotheses to restrict the analysis to a predefined set of regions of interest (ROIs). In particular, it generally requires the choice of an atlas (structural or functional) to identify the ROIs and extract the average time series, which are then fed into a linear correlation analysis. These results can be then represented as FC matrices (ROI-to-ROI connectivity), where each entry denotes links in terms of correlation. While most of the studies tend to focus on a single FC approach, the two methods may reveal complementary information about the connectivity patterns especially in clinical populations, such as voxels of altered FC in a given network of interest (from ICA) as well as the affected connections between distinct brain areas (from seed-based).

In terms of ASL-based FC, few previous works applied the abovementioned connectivity measures to demonstrate that FC can be reliably detected by perfusion and to investigate its relationship with BOLD-based perfusion. Jann et al. (2013) demonstrated the capability of both ICA and seed-based analysis to identify FC networks in ASL data during a motor task paradigm on healthy controls. Noteworthy, beside FC, ASL enabled to quantify the level of activity as CBF within a specific network, which would not have been feasible using BOLD-based measures (Jann et al., 2013). Recently, Dai et al. (2016) applied ICA to separate resting-state signals in an ASL dataset of healthy subjects, recovering some of the main RNSs reported in literature for BOLD fMRI (Dai et al., 2016).

In summary, these connectivity analyses allowed to obtain novel insights and demonstrated to hold great potential as a tool for probing brain functionality in controls and in several brain pathologies, including epilepsy. The latter is a neurological condition characterized by recurrent and spontaneous seizures. About 30% of patients do not respond to anti-epileptic drugs (drug-resistant epilepsies) with the majority of them having temporal lobe epilepsy (TLE) (Bernhardt et al., 2015). These patients require a more specific assessment, which usually involves the combination of several imaging modalities, to guide surgical resection of the presumed epileptogenic area. To this end, over the past years there has been a shift from localization (detection of the epileptogenic area) to connectivity (understanding the connections of the altered area with the rest of the brain and thus the seizure spread), by considering epilepsy as a “network disorder” (Centeno and Carmichael, 2014; Bernhardt et al., 2015; Storti et al., 2017a). Several previous studies relying on BOLD fMRI and electroencephalography (EEG) reported alterations of resting-state FC measures in TLE mainly involving the epileptogenic network (temporal and mesiotemporal structures) together with selective RSNs as the default mode network (DMN), attention and sensory processing networks (Bernhardt et al., 2015). In epilepsy, ASL has been used as a quantitative measure of CBF to localize areas of interictal hypoperfusion/ictal hyperperfusion related to the focus (Wolf et al., 2001; Boscolo Galazzo et al., 2015, 2016). However, in light to this new notion of epilepsy as a “network disorder”, ASL becomes central for assessing perfusion-based connectivity as it relies on signals that are more linked to the true neural activity and can provide a more direct and quantifiable assessment of this pathology (Raichle, 1998). To the best of our knowledge, no previous studies have yet investigated ASL-based FC in epilepsy which deserves further investigations.

Therefore, the aim of this work was twofold. First, we aimed at assessing the effect of ICA-based noise reduction on temporal and spatial ASL analyses, focusing in particular on the impact of the cleaning procedure on FC measures in order to build on a reliable basis. Then, once the optimal pipeline for ASL-based FC was identified in controls, the second step consisted in the assessment of how ASL can be applied for connectivity mapping in a homogeneous group of drug-resistant right TLE patients when compared to an age/gender- matched group of controls. Two complementary FC analyses were applied to ASL data, in order to derive a complete picture of epileptic brains at voxel-wise and region-wise levels. In particular, we aimed to:

(1) Determine voxel-wise between-group differences for each RSN and detect disrupted areas within each network (from ICA analysis).
(2) Quantify ROI-wise between-group FC changes and assess disrupted functional connections between brain regions (from ROI-to-ROI analysis).

## Materials and Methods

### Population

Ten consecutive patients with drug-resistant TLE (PT, mean age = 33.6 ± 8.6 years, 5 males), undergoing pre-surgical assessment, were enrolled. The inclusion criteria were: (1) refractory right TLE; (2) well-defined localization of seizure-onset through EEG and video-telemetry (ictal/interictal); (3) negative anatomical MRI images. Exclusion criteria were as follows: alterations on structural images, general contraindications, presence of other neurological symptoms, seizure onset located outside the temporal lobe structure, diffuse or bilateral epileptic spikes, and pregnancy. Ten age/gender-matched healthy controls (HC, mean age = 32.7 ± 6.8 years, 5 males) with no history of neurological/psychiatric symptoms were also recruited as control group.

The study was approved by the Northeast-Newcastle and North Tyneside 1 Research Ethics Committee and carried out in accordance with the Declaration of Helsinki of the World Medical Association. All subjects gave informed consent prior entering the study. Patients demographics and characteristics are reported in Table 1.

**Table 1:** Clinical profile for the patient population.

Pt n°	Sex	Age	Years since beginning	Type of seizures	Seizures frequency	Relevant history	Current antiepileptic therapy
1	M	19	15	Simple partial with SG	1–2/week (partial), 1–2/month (SG)	Negative	CLZ, OCX, LEV
2	F	41	17	Complex partial	2–3/day	Negative	ZNS, ESL, CLZ
3	M	31	15	Simple partial with SG	2/month, rare (SG)	Negative	LEV, LCM
4	M	23	14	Complex partial	2/day	Negative	VPA, TPM, LEV
5	M	32	8	Complex partial with SG	1–2/month	Negative	OCX, ZNS, VPA
6	F	49	42	Complex partial	2–3/week	Negative	VPA, CBZ, CLZ, ZNS
7	M	40	7	Complex partial	3/week	Negative	CBZ, LTG
8	F	25	12	Complex partial	3–4/day	Negative	LEV, CBZ, VPA, LCM
9	F	50	44	Complex partial	1/day	Negative	LEV, LCM
10	F	33	30	Complex partial	3–4/week	Negative	OCX, TPM, CLZ, CLP

*Gender, age, seizures characteristics, and antiepileptic therapy are reported in table for each patient. In all cases, the electro-clinical diagnosis from the multi-disciplinary team reported a right temporal lobe epileptic focus. SG, secondary generalization; CLZ, Clobazam; OCX, Oxcarbazepine; LEV, Levetiracetam; ZNS, zonisamide; ESL, Eslicarbazepine; LCM, lacosamide; VPA, valproate; TPM, topiramate; CBZ, Carbamazepine; LTG, Lamotrigine; CLP, Clonazepam.*

### Image Acquisition and Protocol

Imaging was carried out on a 3T PET/MRI scanner (Biograph mMR, Siemens Healthcare, Erlangen, Germany) with a 16-channel head and neck coil. ASL data were acquired while subjects were in resting state and were instructed to lie still in the scanner, to keep their eyes closed, and not to fall asleep during the whole scanning time. The manufacturer’s pulsed PICORE (proximal inversion with a control for off-resonance effects) ASL sequence with Q2TIPS (QUIPSS II with Thin-slice TI1 Periodic Saturation) and 2D-echo planar imaging readout was used (voxel size: 3.6 × 3.6 × 5 mm^3^; gap: 1 mm; 19 slices; TI1/TIs/TI2: 800/1200/1800 ms; TR/TE: 2860/17 ms). Four single-session ASL runs of 100 volumes each were acquired 5 min apart one from the other, for a total of 200 Control/Label pairs plus a calibration scan (M_0_) with long TR. A 3D T1-weighted MPRAGE anatomical scan was also acquired for each subject (voxel size: 1.1 × 1.1 × 1.1 mm^3^; 208 sagittal slices, TR/TE: 2000/2.92 ms). In addition, standard MRI sequences [e.g., T2-weighted image and 2D FLAIR (coronal and axial planes)] were acquired in patients as part of the clinical epilepsy protocol.

### Pre-processing and Cleaning Approaches on HC Data

As preliminary step, in this work we aimed at systematically analyzing the influence of the individual cleaning steps of ASL data by relying on ICA-based artifact removal. Only HC data were employed in this first phase, in order to identify the optimal processing in physiological conditions and avoid any possible bias due to the pathology. The individual steps for the analysis of ASL data were carried out using FSL 5.0.9 (FMRIB, Oxford, United Kingdom). Of note, the whole ASL time course was considered, as suggested for task-based ASL data (Mumford et al., 2006), rather than performing all the analyses on the Control-Label subtraction or on the CBF images. While this no-differencing method has been proven to provide increased sensitivity for localizing brain activations, it has only recently started to be investigated in resting-state studies as well with promising results (Storti et al., 2017b; Hao et al., 2018). In this way, we can rely on a high temporal resolution and high-frequency content that is fully retained in the undifferenced data. In particular, two cleaning pipelines were considered for each subject:

(1) *Basic pipeline*: only standard pre-processing steps were applied to each single-session ASL run separately. These included head motion correction with MCFLIRT, non-brain tissue removal with brain extraction tool (BET), spatial smoothing (6-mm FWHM Gaussian kernel), and high-pass temporal filtering (0.01 Hz) for removing slow drifts.
(2) *Advanced pipeline*: each single-session ASL run, minimally pre-processed with the basic pipeline, underwent single-subject probabilistic ICA to decompose the data into several ICs. The MELODIC tool (Beckmann and Smith, 2004) with automatic dimensionality estimation was used for performing the ICA-based artifact removal. In particular, each IC was visually inspected and hand-labeled in order to identify those ICs corresponding to artefactual processes in the data. The manual classification of a component into signal or noise was performed following well-established criteria in literature (Griffanti et al., 2014; Salimi-Khorshidi et al., 2014). In particular, three complementary pieces of information were evaluated, as described in (Griffanti et al., 2017): the IC spatial map, its time series, and its power spectral density. IC maps were classified as signals of interest if they showed well-defined gray matter clusters, were characterized by predominantly low-frequency power spectra (<0.1 Hz) and had similar patterns to those described in literature (Beckmann et al., 2005; Smith et al., 2009). It is important to note that the baseline perfusion component, displaying the typical zig-zag pattern (frequency range of 1/TR), was always retained although above the general 0.1 Hz frequency cut-off (Hao et al., 2018). The noise components were finally regressed out from the basically pre-processed ASL data, resulting in four ASL runs with advanced cleaning per subject.

To investigate the impact of cleaning procedures on the ASL signal as well as on the most common FC outcomes, the efficacy of the two procedures was tested through time series and spatial map analyses. In addition, we evaluated the effects of the ICA cleanup on CBF estimates, in order to fully assess the impact of the denoising on the dual information that ASL can provide (connectivity and perfusion). Regarding time series analysis, a global measure of temporal SNR (tSNR) was firstly calculated for each ASL run separately. The tSNR image was derived dividing the mean across time by the standard deviation over time. This was then eroded to exclude possible edge effects, and the median across space was retained for further analyses. A paired sample *t*-test was applied to compare the tSNR values between the two pipelines (*p* < 0.05, Bonferroni-corrected for multiple comparisons). In addition, a one-way analysis of variance (ANOVA) for repeated measures was performed on the tSNR values of the four ASL runs, considering separately the basic and advanced pipelines. *Post-hoc* paired sample *t*-tests were also applied wherever appropriate.

Two additional measures were included in the comparison to support the tSNR metric: loss of temporal degrees of freedom (tDoF) (Pruim et al., 2015; Dipasquale et al., 2017) and %ΔSTD maps (Khalili-Mahani et al., 2013; Griffanti et al., 2016; Carone et al., 2017). Regarding the loss of tDoF associated with the two cleaning pipelines, this was assessed to evaluate their potential impact on the statistical power (Pruim et al., 2015). For each run, the total number of volumes was considered to be the total number of tDoF initially available in the data. We then calculated how many ICs were regressed out from the data (for each subject and run) and every IC that was removed was considered as a single tDoF. The lost tDoF was finally expressed as a percentage of the total available tDoF.

The %ΔSTD maps (Khalili-Mahani et al., 2013) were computed to quantify the percentage of the voxel-wise temporal fluctuation amplitude of the minimally pre-processed data (basic) that is suppressed by the advanced cleaning. This map was calculated for each subject (separately for each run) as follows:

$$\%\Delta {\mathrm{STD}}_{\mathrm{map}}=100\cdot \frac{\mathrm{STD}({\mathrm{img}}_{\mathrm{basic}})-\mathrm{STD}({\mathrm{img}}_{\mathrm{advanced}})}{\mathrm{STD}({\mathrm{img}}_{\mathrm{basic}})}$$

where STD is the standard deviation of each voxel over the acquired ASL volumes.

The %ΔSTD maps were then spatially normalized to the 2-mm MNI152 standard space (FNIRT) and used to build, for each of the four runs, a probability map of areas where %ΔSTD > 25% across subjects. This would highlight in which areas the ASL variance was more frequently reduced across subjects by the advanced cleaning (Griffanti et al., 2016; Carone et al., 2017).

Regarding spatial map analysis, in absence of a ground-truth of the neural signal, our working assumption was that the cleaning of the data would enhance the similarity and the spatial correlation of each IC to the corresponding reference template (for ICA and RSNs) (Griffanti et al., 2016). For ICA, a multi-session temporal concatenation analysis as implemented in MELODIC was run for each subject and pipeline, after having non-linearly registered all the ASL data to the 2-mm MNI152 standard space (FNIRT). The resulting IC maps were converted to *z*-statistic maps and threshold at *z* = 3. The most common RSNs described in literature were identified at the individual level and then compared to the corresponding well-known template from Smith et al. (2009) encompassing 20 RSNs. Of note, this template has been considered in this study despite being derived from BOLD fMRI as no reference RSN templates derived from ASL are currently available in literature. Indeed, as ASL is still not widely used and accepted in the FC field, a technique-specific RSN template is not available yet, precluding the possibility to compare our results with a common ASL reference from literature. In our opinion, this template can be reasonably considered the gold standard for the assessment of ASL-based ICA maps as well, as we aimed to demonstrate that ASL can extract similar RSNs to those reported in literature in BOLD studies (Dai et al., 2016). The spatial similarity/overlap between each IC map (derived from the two pipelines) and the corresponding BOLD template was determined using the Dice Similarity Coefficient (DSC) (Dice, 1945). This index compares the number of common voxels as follows: DSC (A, B) = 2(A∩B)/(A + B), where A and B are the two IC maps. In addition, the level of spatial cross-correlation (*r*-value) was assessed for each RSN using the *fslcc* tool in FSL. For each RSN independently, the DSC and *r*-values calculated from the basic and advanced pipelines were statistically compared through paired sample *t*-tests (*p* < 0.05).

A group-ICA was also performed by temporally concatenating all the subjects/sessions and the resulting group RSNs were spatially compared to the reference template in terms of DSC and *r*-values as above. This step aimed at providing further information about the impact of the cleaning procedures on the RSNs estimated at both single and group level.

Finally, regarding the CBF analysis, the ASL volumes filtered with both basic and advanced pipelines were pairwise subtracted and averaged to obtain perfusion-weighted images. These maps were quantified into CBF (ml/100 g/min) applying the general kinetic model (Buxton et al., 1998) as follows:

$$\mathrm{CBF}=\frac{6000\cdot \text{λ}\cdot \Delta M\cdot e^{\frac{\mathrm{TI}2+(n-1){\mathrm{slice}}_{\mathrm{time}}}{T_{1B}}}}{2\cdot \text{α}\cdot \mathrm{TI}1\cdot M_{0t}}$$

where *λ* is the brain–blood partition coefficient (0.9 mL/g), ΔM represents the difference images (perfusion-weighted maps), TI1 and TI2 are the sequence time parameters described above, *n* is the slice number, *slice_time_* is the time taken to acquire each single slice (∼55 ms), *T*_*1B*_ is the longitudinal relaxation time of blood (1650 ms at 3T), α is the inversion efficiency (0.95 for pulsed ASL) (Alsop et al., 2015), and *M*_*0t*_ is the tissue equilibrium magnetization (voxel-wise estimated from the calibration scan).

For each subject, these CBF maps in native space were spatially normalized into the 2-mm MNI152 standard space, using the transformations previously estimated. Considering all controls, the maps resulting from the two pipelines were statistically compared on a voxel-wise basis (Wilcoxon’s rank sum test, *p* < 0.05 with false discovery rate correction for multiple comparisons) and group CBF maps were derived separately for the two approaches. To provide representative CBF values, we calculated the mean CBF across gray and white matter (GM, WM) for each subject in native space. To do this, each subject’s structural image was segmented to obtain tissue probability maps for GM and WM, which were then thresholded at 0.9 and binarized. These masks were back-projected in ASL space by inverting the linear registration from ASL to the corresponding T1-weighted image (Boundary-Based Registration, ASL-to-T1). Additionally, we computed the ratio of mean CBF in GM over WM as a measure of contrast ratio for each pipeline (Vidorreta et al., 2013).

### FC Analysis on Patients and Controls: ICA and RSN (Within-Network Connectivity)

The optimal pipeline resulting from the previous evaluations on the HC group was applied to process all the PT data before performing FC analyses and group comparisons.

In order to extract the different RSNs, group-ICA using MELODIC in FSL was performed, temporally concatenating the functional images of all subjects/sessions in the temporal domain to create a single 4D dataset. In particular, each cleaned dataset was firstly linearly registered to the corresponding structural image using FLIRT with Boundary-Based Registration (Greve and Fischl, 2009) and then spatially normalised to the 2-mm MNI152 standard space using non-linear registration (FNIRT). This concatenated dataset was decomposed with automated dimensionality estimation, leading to IC maps (2 × 2 × 2 mm^3^) reflecting both the RSNs of interest as well as residual noise components.

In order to recover the individual RSNs and assess within-network group differences, the whole output from the group-ICA was used as input template in a dual regression algorithm. Indeed, this approach allows to detect voxel-wise group differences in the FC of the different RSNs as well as to identify subject-specific networks based on those identified at the group level (Beckmann et al., 2005; Filippini et al., 2009). This involved two subsequent steps: spatial and temporal regression. First, the full set of IC spatial maps from group-ICA was linearly spatially regressed against the single-subject pre-processed data, providing a set of subject-specific time series (one per each component). Then, these individual time series were variance normalized and temporally regressed against the corresponding pre-processed data, converting each time series to an individual IC spatial map. Note that, as for each subject multiple ASL runs were available, the average across the four session maps was performed to get a single IC per participant to be used for the subsequent statistical analysis. For each component of interest, a non-parametric permutation testing using the randomize feature in FSL was conducted (5,000 permutations) in order to statistically compare the IC spatial maps and identify between-group differences at the voxel-wise level. The resulting group difference maps were thresholded using a threshold-free cluster enhancement (TFCE) technique and a corrected *p* < 0.05 for multiple comparisons (family wise error, FWE) (Nichols and Holmes, 2002). Group maps were also obtained for HC and PT performing a one-sample *t*-test on the subject-specific spatial maps derived for each component as output of the second stage of dual regression, calculating the corresponding *z*-map and applying a mixture model correction (*z* = 3 as threshold) (Filippini et al., 2014; Griffanti et al., 2014).

### FC Analysis on Patients and Controls: ROI-to-ROI Correlation Matrices (Between-Region Connectivity)

We developed in-house MATLAB scripts to perform ROI-based FC analysis on subject-specific ASL cleaned data. Indeed, while dual regression ICA allows investigating between-group differences in the co-activity of each RSN, complementary information about possible changes in inter-region FC can be derived from ROI-to-ROI connectivity analysis. Regarding the brain parcellation, ROIs were defined by using the individual RSNs as masks to extract the time series. Noteworthy, we are not dealing with the time course of the whole map *per se*, but with the signals from the different regions composing each IC. Specifically, for each subject, the networks of interest were subdivided into distinct spatially contiguous regions, representing the main nodes of each RSN, by applying the cluster tool in FSL on the individual spatial maps from the second stage of dual regression (using *z* = 3 as threshold). To verify the reliability of the ICA-based ROIs, the results were visually checked according to the well-known literature findings and the main clusters from the BOLD IC template (Smith et al., 2009) were further used as additional reference to confirm the identification of the network nodes. For example, for the DMN eight main ROIs were depicted in all subjects, consistently with previous works (Pievani et al., 2017).

Then, for each subject, the four cleaned and spatially normalized ASL runs were voxel-wise demeaned, variance normalized, and temporally concatenated resulting into time series of 400 volumes length. For each region, a representative mean time course was extracted averaging the time series of all the voxels within the area. A symmetric connectivity matrix was derived for each subject calculating the Pearson correlation coefficient between pairs of nodes. Matrices were compared between the two groups through non-parametric permutation testing (5,000 permutations) with a significance threshold of 0.05 (FWE-corrected) (Nichols and Holmes, 2002). Given the exploratory nature of the study, the statistical results uncorrected for multiple comparisons are also reported.

## Results

### Effect of Cleaning on ASL HC Data

The basic and advanced pipelines were successfully employed to clean all the ASL data of the HC group. Our results revealed that an advanced cleaning based on ICA artefactual removal could improve both the signal properties of the ASL signals (in terms of tSNR combined with lost tDoF and ASL variance reduction) as well as the spatial similarity of the different spatial components to the literature templates (in terms of DSC and *r*-values). Moreover, results revealed that the impact of ICA on CBF values was minimal, allowing to preserve the important baseline perfusion content.

The ICA cleanup removed several components of no-interest (Supplementary Table S1). On average, the following percentages of components removed during the denoising were found (mean ± standard deviation values across subjects): 23.1 ± 7%, 24.4 ± 7.5%, 22.9 ± 7%, and 21.4 ± 5% for the first, second, third, and fourth run, respectively.

**FIGURE 1:**
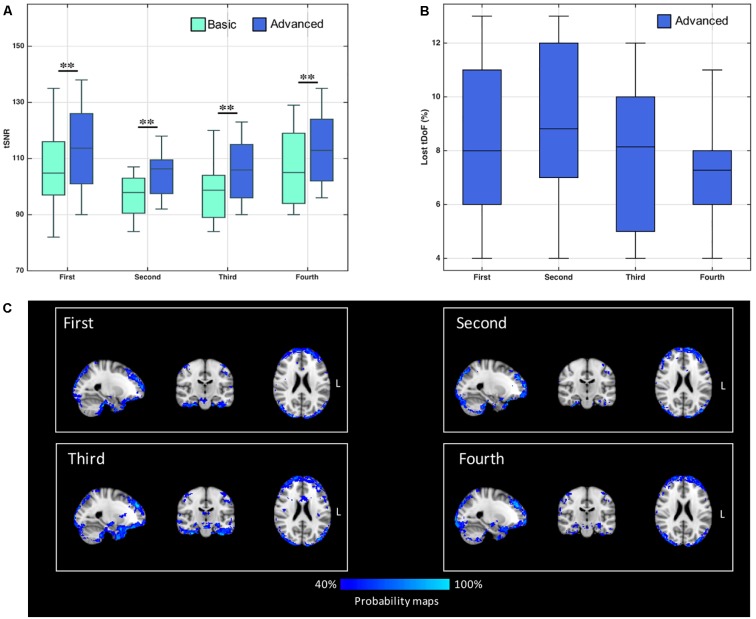
**(A)** Distribution of temporal signal-to-noise ratio (tSNR) values derived from control’s data, with both basic and advanced cleaning pipelines. The four ASL runs were considered separately (^∗∗^*p* < 0.01, Bonferroni-corrected for multiple comparisons). **(B)** Loss of temporal degrees of freedom (tDoF) for each of the four runs. Only the advanced pipeline was considered, as no lost tDoFs were present in the case of the basic cleaning. **(C)** Spatial pattern of changes in ASL signal standard deviation after using the advanced pipeline. The probability maps, representing areas where the variance was reduced more frequently in controls, are reported for each run separately. These maps represent for each voxel the percentage of subjects with %ΔSTD > 25%.

The median tSNR values for the controls with the two cleaning options are reported in Figure 1A. Considering separately the four ASL runs, the tSNR was significantly higher after cleaning with the advanced pipeline (*p* < 0.01, Bonferroni-corrected) in all cases. As a side note, the values were comparable across the sessions for both pipelines, with no statistically significant differences [basic: *F*(3,36) = 0.81, *p*-value = 0.49; advanced: *F*(3,26) = 0.99, *p*-value = 0.41].

The loss of tDoF related to the advanced pipeline was similar across the four runs, with an average value of 8.0 ± 2.7% across subjects and runs (Figure 1B). The probability maps showing the distribution of ASL fluctuation reduction across subjects are reported in Figure 1C. By relying on the ICA-based cleanup, the highest reduction of fluctuations was localized at brain edges, where motion artifacts are predominant, and in areas corresponding to blood vessels, as the posterior cerebral artery, the middle cerebral artery, and pontine arteries. Similar patterns were found across the four single runs. An average decrease of 33% in the ASL signal variance was found when the advanced pipeline was applied.

**FIGURE 2:**
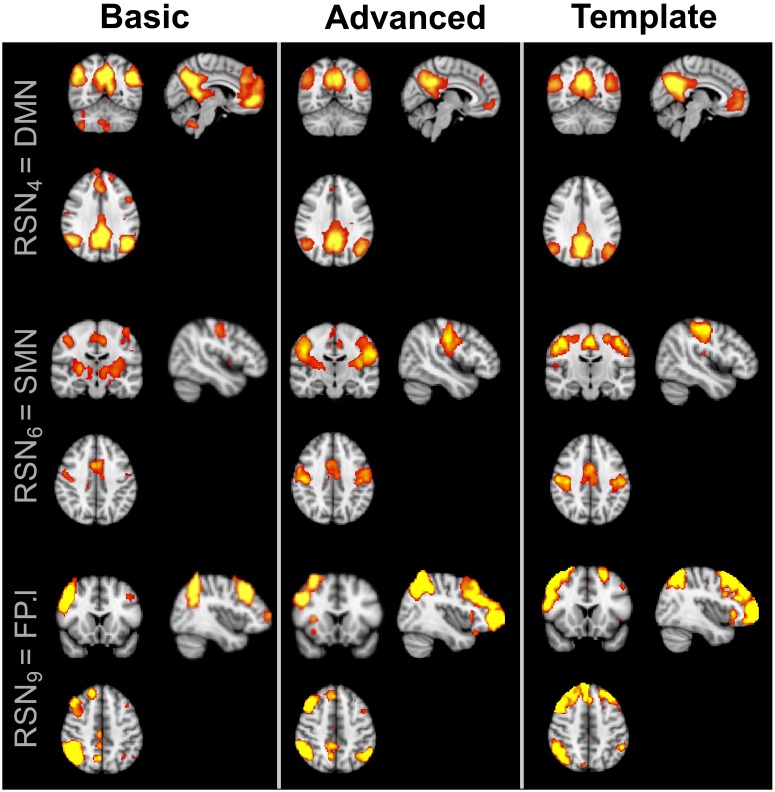
Example of three resting-state networks (DMN, SMN, and FP.l) derived from separate healthy subjects by applying the two different cleaning pipelines to the ASL datasets. The corresponding BOLD template from literature is also reported for reference. All the component maps were thresholded at *z* = 3.

**FIGURE 3:**
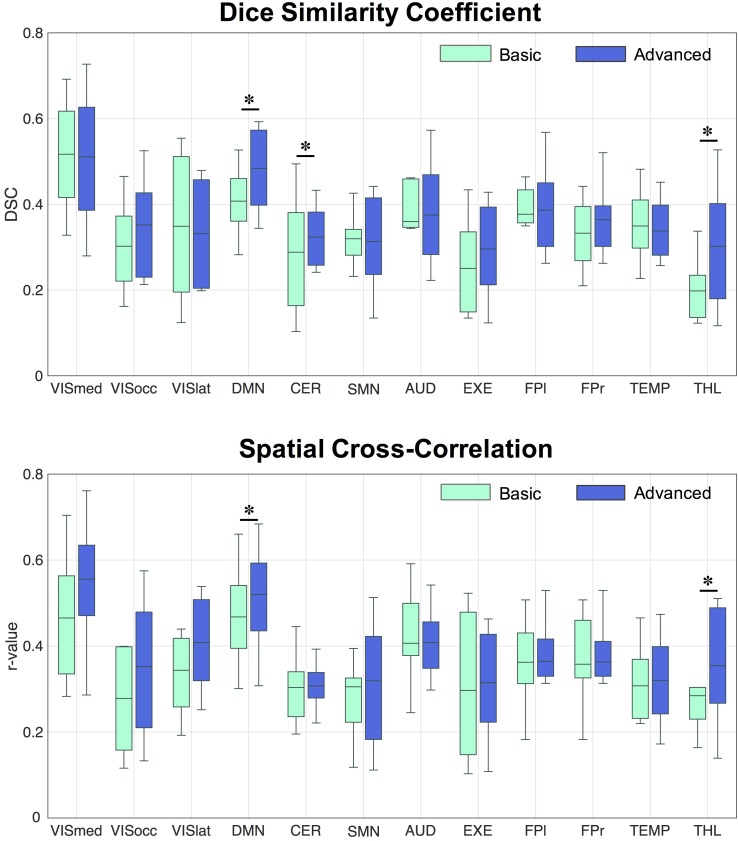
Distribution of Dice similarity coefficient and spatial cross-correlation values across healthy controls. The two indices were calculated for each spatial component derived from the data cleaned with the basic and advanced pipelines, respectively, by comparing the network of interest to the corresponding template component. ^∗^Statistically significant differences, *p* < 0.05.

Regarding the spatial map analyses, the ICA decompositions on basic and advanced cleaned data revealed 12 common RSNs in all subjects: three visual networks (medial [VISmed], occipital [VISocc], and lateral [VISlat]), DMN, cerebellum (CER), sensorimotor (SMN), auditory (AUD), executive (EXE), left/right frontoparietal (FP.l, FP.r), temporal (TEMP), and thalamus (THL). Examples of how the extraction of the RSNs at the individual level is influenced by the specific cleaning procedures are reported in Figure 2 for three representative networks (DMN, SMN, and FP.l) derived from separate controls. Visually evaluating the IC maps, a good similarity with the BOLD template was appreciable in all cases, although a closer match was found when the ASL data were processed with the advanced cleaning. This is particularly evident in the case of the SMN example of Figure 2, where the right-left primary motor areas were only partially recovered with the basic pipeline and a significant contamination from the subcortical regions was found. When these evaluations were quantified in terms of spatial similarity with DSC, the results consistently showed, across subjects, a good level of overlap with the corresponding reference networks (Smith et al., 2009). This is visible in the boxplots of Figure 3 (top) reporting the distribution of individual DSC values calculated at single-subject level for all the identified networks. This overlap further increased with the application of the advanced pipeline in all cases. In particular, for three RSNs (DMN, CER, and THL) the similarity was proved to be significantly higher when the advanced cleaning was applied to the ASL data (*p* < 0.05). This trend was further confirmed by the spatial cross-correlation analyses, summarized in Figure 3 (bottom), where we found for all the twelve RSNs an overall mean *r*-value >0.25, which is generally considered a valuable cut-off value for classifying a good component from BOLD fMRI data (Smith et al., 2009). The DMN and THL resulted to be the networks with significantly higher values after the advanced cleaning (*p* < 0.05) also in terms of spatial correlation.

When assessing the impact of the processing pipeline on the group-ICA results, consistent results as for the single-subject ICA were found, showing increases in spatial overlap/correlation with the advanced pipeline (Table 2). While some RSNs only partially benefited from the ICA-based processing (e.g., VISocc, CER, FP.l, and FP.r), for other networks, as VISmed, DMN, AUD, EXE, and THL, the DSC/*r*-values were markedly higher after the advanced cleaning. This was visible also in the SMN network, which further confirmed the difficulties in its extraction already found at the individual level. Considering the complementary information provided by DSC and *r*-value, EXE and VISmed were overall the RSNs with the minimum and maximum similarity to the BOLD template, respectively, for both basic and advanced cleaning.

**Table 2:** Group resting-state networks (RSNs) and similarity indices.

RSN	Dice coefficient		Spatial correlation	
	Basic	Advanced	Basic	Advanced
VISmed	0.646	0.726	0.729	0.752
VISocc	0.313	0.380	0.273	0.344
VISlat	0.590	0.640	0.583	0.609
DMN	0.520	0.661	0.701	0.737
CER	0.257	0.290	0.184	0.208
SMN	0.200	0.481	0.224	0.449
AUD	0.396	0.612	0.447	0.590
EXE	0.178	0.331	0.185	0.287
FP.l	0.501	0.513	0.385	0.492
FP.r	0.449	0.497	0.391	0.453
TEMP	0.409	0.491	0.388	0.464
THL	0.255	0.673	0.214	0.673

*Dice similarity coefficient (DSC) and spatial cross-correlation (*r*-value) values calculated between each RSN and the corresponding BOLD template are reported for both basic and advanced pipelines.*

**FIGURE 4:**
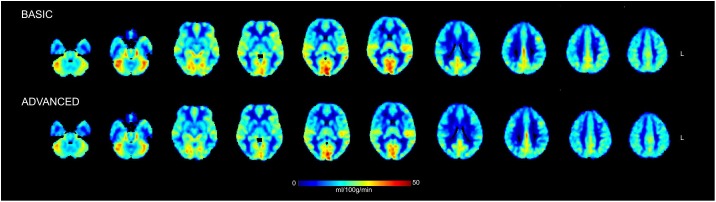
Mean cerebral blood flow (CBF) maps in physiological units (ml/100 g/min) calculated across the group of healthy controls from the ASL data cleaned with the basic and advanced pipelines. Some representative slices of interest in the 2-mm MNI152 standard space are reported in radiological convention. No statistically significant changes were detected between the CBF maps derived from the two pipelines when compared on a voxel-wise basis.

Finally, similar CBF maps for both basic and advanced pipelines were found (Figure 4). The qualitative similarity between maps was confirmed by the Wilcoxon’s rank sum test (*p* < 0.05, corrected) which revealed no statistically significant changes pre/post ICA-based cleanup. Considering as representative measures the mean values over GM and WM, CBF was reduced of about 2.6% in GM (from 35.1 ± 4.5 ml/100 g/min to 34.2 ± 4.5 ml/100 g/min) and of about 5.1% in WM (from 13.5 ± 2.7 ml/100 g/min to 12.8 ± 2.2 ml/100 g/min). The GM-to-WM contrast revealed a slight increase with the advanced pipeline (from 2.6 to 2.7).

Based on all these results, the advanced cleaning pipeline was chosen for all the subsequent analyses and processing of patient’s data.

### FC Analysis in Epilepsy Patients and Controls – ICA and RSNs

**FIGURE 5:**
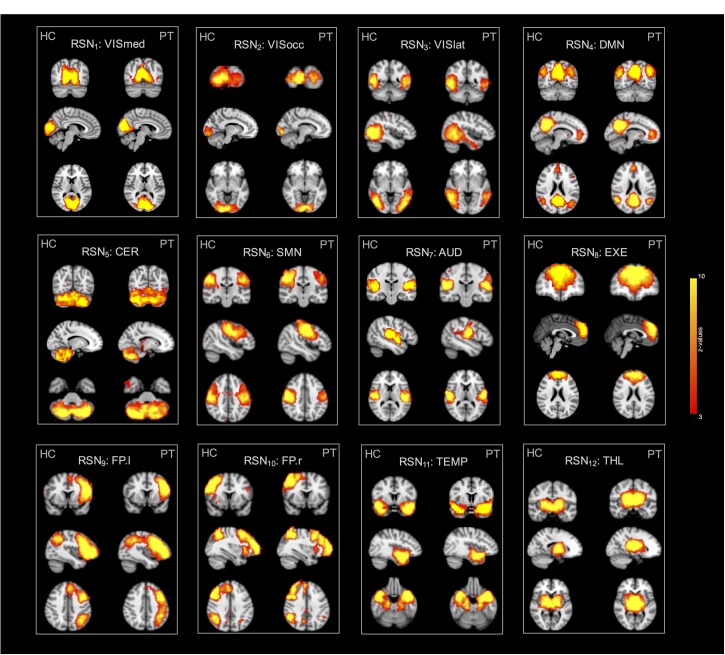
Twelve resting-state networks (RSNs) of interest derived from the control and patient group analyses. All spatial maps were converted to *z*-statistic images and thresholded at *z* = 3. Each map was superimposed to the 2-mm MNI152 standard space and shown in radiological convention.

**FIGURE 6:**
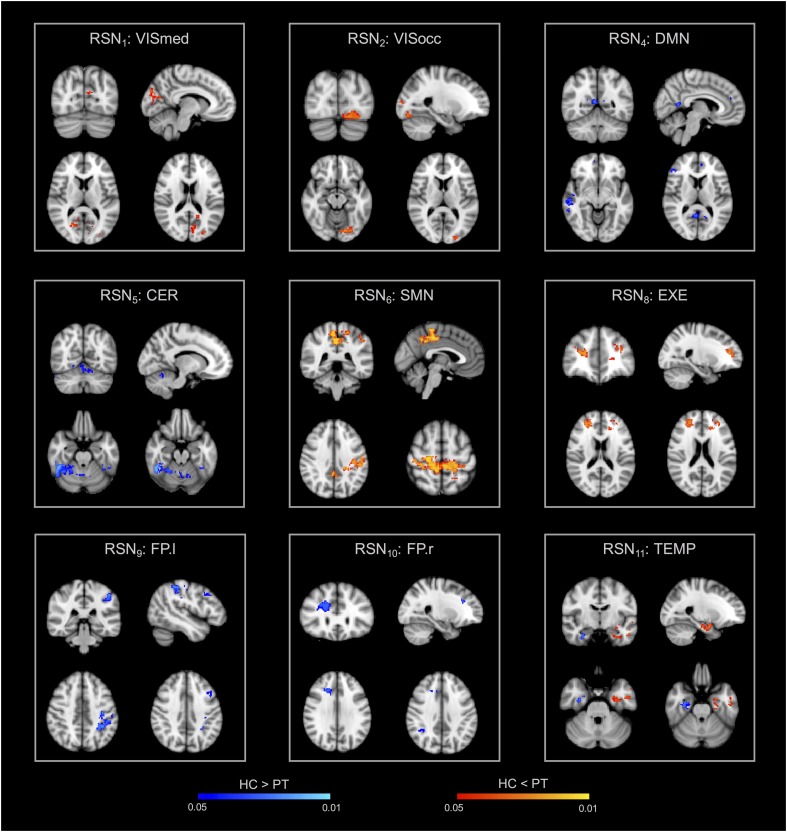
Within-network functional connectivity (FC) differences between patients and controls resulting from the permutation testing on the dual regression outputs. Clusters of significant difference (*p* < 0.05, FWE-corrected with TFCE) are overlaid on the MNI152 standard space and shown in radiological convention. In particular, areas featuring decreased FC in patients are reported with the blue-light blue colormap (HC > PT), while increased FC patterns in patients are decoded with the red-yellow colormap (HC < PT).

The group-ICA decomposition performed on the temporally concatenated HC and PT data produced 43 ICs. Out of these, 32 were visually classified to be noise-related artifacts representing mainly head motion, scanner/hardware drifts, signals from white matter, and cerebrospinal fluid. These components were discarded from further analyses, leaving 12 RSNs of interest retained for the subsequent dual regression analysis. In particular, the same networks already found in the HC group (at both subject- and group-level) were here recovered. The corresponding group IC maps, thresholded at *z* = 3, are reported in Figure 5 for HC and PT separately. While all the ASL-based RSNs were visibly consistent with the canonical BOLD networks reported in literature, also for the epilepsy patients, between-group differences were identified in the structure of some ICA networks when statistically compared. Regarding the voxel-wise analysis with permutation testing and a corrected threshold (*p* < 0.05, FWE), areas of decreased connectivity only were found in four RSNs in the PT group compared to HC. The most prominent changes were detected in the CER, DMN, and FP.l (Figure 6 and Table 3). Most of the significant clusters were located in the ipsilateral hemisphere, as clearly visible for the DMN and CER. Conversely, the statistical analysis revealed voxels of significant FC increase in epilepsy patients with respect to HC in the VISmed, VISocc, SMN, and EXE (*p* < 0.05, FWE). These clusters were mainly located contralateral to the epileptic focus. For the TEMP network, significant alterations over the temporal lobes (TLs) were found. In particular, areas of increased connectivity were identified in PT in the contralateral hemisphere to the epileptic focus, while the reversed pattern was seen ipsilaterally. Finally, no changes were shown in the remaining networks (Figure 6 and Table 3).

**Table 3:** Clusters of between-group differences in within-network connectivity.

	ROI	Voxels	MAX X (mm)	MAX Y (mm)	MAX Z (mm)	*p*-Value peak
**HC > PT**						
DMN	MTG.r/TL.r	367	60	−22	−10	0.030
	MFG.r	348	54	34	20	0.034
	PCC.r/PL.r	141	6	−54	10	0.029
CER	CER.r	2478	48	−50	−28	0.048
	CER.l	1254	−36	−40	−34	0.050
FP.l	SPL.l/PL.l	840	−32	−48	48	0.048
	MFG.l/FL.l	89	−44	14	38	0.050
FP.r	FL.r	460	48	50	10	0.029
	PPC.r	267	48	−56	28	0.048
TEMP	TL.r/pHiPPa.r	791	32	−8	−32	0.042
**HC < PT**						
VISmed	LgG.l/TOF.l	286	−28	−48	−6	0.028
	OFG.r/OL.r	141	26	−68	−10	0.038
	OL.l/PL.l	31	−16	−68	18	0.041
VISocc	OFG.l/OL.l	283	−16	−80	−14	0.017
	LOC.l/OL.l	133	−28	−94	−2	0.042
SMN	PRG.l/pCg.l	2959	−12	−32	40	0.010
	SMC	50	−6	−22	60	0.044
EXE	FL.r/SFG.r	467	26	44	14	0.030
	FL.l/MFG.l	463	−32	44	20	0.038
	PAC/ACC	30	0	46	−2	0.044
TEMP	TL.l/pHiPPa.l	1284	−24	−4	−22	0.041

*The statistical results identified by the permutation testing on the dual regression output are reported for each resting-state network (*p* < 0.05, FWE-corrected with TFCE). In particular, clusters of within-network decreased connectivity in patients compared to controls (HC > PT) as well as those of increased connectivity (HC < PT) were identified. MTG, middle temporal gyrus; TL, temporal lobe; MFG, middle frontal gyrus; PCC, posterior cingulate cortex; PL, posterior lobe; CER, cerebellum; SPL, superior parietal lobe; FL, frontal lobe; PPC, posterior parietal cortex; pHIPP, parahippocampus; LgG, lingual gyrus; TOF, Temporooccipital fusiform gyrus; OFG, orbitofrontal gyrus; OL, occipital lobe; LOC, Lateral occipital lobe; PRG, precentral gyrus; pCg, postcentral gyrus; SMC, supplementary motor cortex; SFG, superior frontal gyrus; PAC, paracingulate gyrus; ACC, anterior cingulate cortex; l, left; r, right.*

### FC Analysis in Epilepsy Patients and Controls – ROI-to-ROI Correlation

**FIGURE 7:**
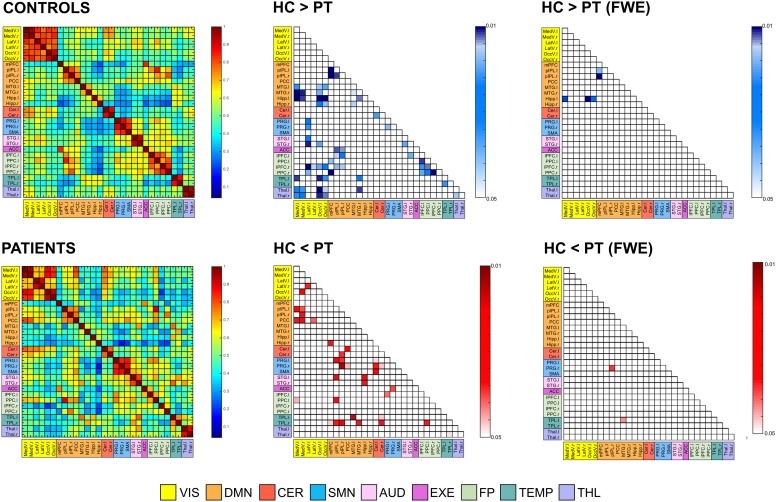
Functional connectivity (FC) matrices averaged across all subjects for the two groups (controls [HC] and patients [PT]). Thirty regions of interest were extracted from the 12 resting-state networks, and were sorted in the matrices according to their belonging network. Regions are the row and column indices of the FC matrices, and the corresponding matrix element provides the color-coded Pearson correlation coefficient. The significant FC links (*p* < 0.05, both without correction for multiple comparisons and FWE-corrected) are also reported for the control vs. patient analysis. Each element of these matrices provides the color-coded *p*-value of the statistical differences, as indicated by the colorbar (blue: HC > PT; red: HC < PT). Only the lower triangular part of the matrix, i.e., excluding self-connections and redundant connections, is shown due to the symmetry of the FC matrix. MedV, medial visual; LatV, lateral visual; OccV, occipital visual; mPFC, medial prefrontal cortex; pIPL, posterior inferior parietal lobule; PCC, posterior cingulate cortex; MTG, middle temporal gyrus; Hipp, hippocampus; Cer, Cerebellum; PRG, precentral gyrus; SMA, supplementary motor area; STG, superior temporal gyrus; ACC, anterior cingulate cortex; lPFC, lateral prefrontal cortex; PPC, posterior parietal cortex; TPL, temporal lobe; THL, Thalamus; l, left; r, right.

The 12 RSNs identified at the group-level were divided into a total of 30 different ROIs, representing the main nodes of each network, and were used to extract the signals to calculate the between-areas FC measures. Figure 7 displays the correlation-based FC values between ROIs, averaged over subjects in each group and expressed as connectivity matrices. The similarity between HC and PT FC matrices was high, as confirmed by the 2D spatial correlation value (*r* = 0.83). However, selective alterations in specific connections were identified when the pairwise ROI correlations were statistically compared. Supra-threshold values resulting from the permutation testing and corresponding to significant correlation differences between groups are also displayed in Figure 7, for both *p* < 0.05 without correction for multiple comparisons and corrected with FWE. When considering the uncorrected *p* < 0.05, several links with diminished FC in PT compared to HC were identified (64/435, 14.7%). Out of these, 22/64 (34.4%) represented altered intra-hemispheric connections (13/64 and 9/64 were located contralateral and ipsilateral to the epileptic focus, respectively), while 28/64 (43.8%) were inter-hemispheric links featuring HC > PT patterns. In addition, 8/64 (12.5%) were inter-hemispheric links connecting the same region in the two hemispheres, as the right-left TL, the right-left THL or the right-left precentral gyrus (PRG). Of note, more than 50% of the altered connections involved one of the visual areas of both hemispheres, as visible in Figure 7.

Considering the reversed pattern (HC < PT), increased FC values were found in the PT for 35/435 links (8.1%). Out of these, the large majority corresponded to intra-hemispheric connections, located both in the right and left hemisphere, resulting in a total of 17/35 (48.6%) altered FC links. Conversely, only 9/35 (25.7%) were inter-hemispheric connections.

Noteworthy, patients resulted to have within-network connections with decreased FC in several RNS, for example within DMN and FP, while the increased connectivity patterns involved mainly the between-network connections. In addition, several connections to the TLs resulted to be altered, with the right TL featuring increased FC with several DMN areas, while the left TL revealed mainly decreased FC with visual areas as well as with other temporal structures.

When correction for multiple comparisons was applied (FWE), few connections survived the thresholding (Figure 7). In particular, the links between the left Hippocampus and visual areas as well as few within-DMN links confirmed the strong decreased patterns in PT compared to HC, while two connections, one of those involving the contralateral temporal areas, presented strong increased FC values in PT.

## Discussion

In this study, we report the first comprehensive assessment of FC patterns and modulations in drug-resistant TLE patients by relying on ASL signals. In addition, we investigated the effect of cleaning procedures on ASL data acquired on HC, in order to identify possible confounding factors for FC analysis and define the optimal pre-processing pipeline in physiological conditions. Our results demonstrated that a more advanced pipeline based on ICA noise removal leads to higher tSNR values, and to a reduction of ASL signal variance particularly evident over problematic areas, as brain boundaries or over blood vessels, with a minimum impact on the CBF estimates. Moreover, it allows the identification of RSNs with enhanced spatial similarity to the available BOLD template. Having identified the best pipeline, we applied it to a dataset of right TLE patients to investigate FC changes in this “network disease” at the voxel (ICA) and ROI level (ROI-to-ROI analysis), and compared them to the HC group. Disrupted brain networks and FC were found in our group of TLE patients. In particular, patterns of increased within-network FC were mainly found contralateral to the epileptic focus, while between-ROI links with increased FC were found ipsilaterally.

FC analyses are currently a useful tool to assess the organization and functionality of the brain networks, along with their possible alterations in patients. FC is defined as the temporal dependencies between neural activity of separate areas and in the resting-state brain can provide a rich set of network features. BOLD-based resting-state FC was firstly demonstrated by Biswal et al. (1995) who showed high correlation between left and right motor areas at rest, suggesting the presence of an intrinsic motor architecture (Biswal et al., 1995). Later on, it was found that different networks of correlated temporal patterns can be recovered at rest, including the DMN which has been shown to be active at rest and impaired in many neurological conditions (Beckmann et al., 2005; Smith et al., 2009). However, BOLD contrast represents only an indirect measure of neural activity, is highly affected by several noise contributions, and often suffers from draining vein contamination. On the contrary, ASL offers a valid alternative as it allows to derive signals directly related to brain perfusion, which is thought to be closely coupled to regional neural activity (Raichle, 1998), and therefore has recently gained interest in the research community for assessing brain resting-state FC. Of note, while almost all these ASL-based FC studies rely on analyses performed on the Control-Label images or on CBF maps directly, in this study we preferred to consider the whole time course, treating the ASL signals as the BOLD ones. In this way, a high temporal resolution and a high-frequency content were available for all the connectivity analyses. This approach has been recommended for task-based analyses (Mumford et al., 2006), but has been scarcely employed for studying resting-state FC with ASL, despite the promising results (Storti et al., 2017b, 2018; Hao et al., 2018).

A key aspect for reliable FC analyses is the choice of the pre-processing applied to the data to recover and separate the signal of interest from the other noise related fluctuations. This aspect has been widely investigated in BOLD-based connectivity and several cleaning pipelines have been developed, mainly based on nuisance regression or on ICA (Pruim et al., 2015). These pipelines result in clean fMRI time series that more accurately reflect the underlying brain fluctuations of interest and reduce possible bias in the subsequent FC analyses (Dipasquale et al., 2017). In nuisance regression-based pipelines, motion parameters are estimated and regressed out (as parameters of no interest) from fMRI signal, together with averaged white matter and cerebrospinal fluid time series. Conversely, ICA-based cleaning methods are tools to decompose the data into signals of interest and structured noise, which are then regressed out from the data. The different components can be either classified by manual labeling or using automatic tools, like ICA-FIX and ICA-AROMA (Salimi-Khorshidi et al., 2014; Pruim et al., 2015) which have been recently developed for BOLD data. In the context of ASL, how noise reduction affects ASL signal properties and FC measures is still largely unknown. Recently, Jann et al. (2016) exploited nuisance regression as a cleaning method of ASL data and investigated its impact on perfusion-based FC in controls and children with autism spectrum disorders. In particular, different pipelines based on nuisance regression were implemented and their effect was investigated in a seed-based analysis aimed at reproducing the DMN. The authors demonstrated a change in FC strength and spatial maps when different nuisance variables were used and highlighted the similarity of ASL results compared to BOLD data (Jann et al., 2016). To date, however, no study has investigated the effect of ICA-based noise reduction on ASL. Only Hao et al. (2018) have reported preliminary results on similar aspects of ICA cleanup for pCASL data. Here, we proposed and assessed the use of ICA as an alternative pre-processing method for ASL data, evaluating its impact on several aspects of the analysis. It is worthy of note, that despite having calculated the tSNR metric, which has been extensively used in literature to compare different pre-processing pipelines, including ICA-based approaches as ICA-AROMA and ICA-FIX (Griffanti et al., 2015, 2016; Dipasquale et al., 2017), this measure might not be suitable for evaluating ICA cleanup on its own. Indeed, as an inherent consequence of the method, increased tSNR values can be found even when a significant number of meaningful components are removed from the data (related to the reduced standard deviation). Therefore, this metric should be treated carefully and assessed jointly with other measures, as the loss of tDoF or the spatial maps of changes in ASL signal standard deviation. The different IC maps were manually classified into signal of interest or noise, as the visual inspection of the components still remains the gold standard (Griffanti et al., 2017), despite being time-consuming and requiring expertise. The validity and reliability of this cleaning method were assessed on both time series, network analysis and CBF estimates. All in all, we were able to demonstrate that ICA-based pre-processing of ASL data can lead to higher tSNR values, good variance reduction (mainly at the brain edges, which are often contaminated by motion artifacts) and improved RSNs recovery, when compared to basic cleaning. Only minor perfusion reductions (not statistically significant) were found after ICA-based denoising. This is in line with the findings shown by Hao et al. (2018), demonstrating a global CBF reduction in both controls (around 2%) and children with sickle cell disease (around 6%).

Having demonstrated that ASL pre-processing using ICA can improve the recovery of RSNs, we applied this cleaning method to a cohort of right TLE patients. In line with the concept of epilepsy as a “network disease,” we were interested in assessing perfusion-based connectivity changes which could highlight pathological mechanisms and modulations in this neurological condition. In particular, we aimed at providing a complete assessment of the main FC analyses, focusing on both ICA and seed-based analyses (ROI-to-ROI) to derive complementary information. On the one hand, ICA can identify areas of increased/decreased connectivity in patient networks compared to controls. On the other, ROI-to-ROI connectivity can highlight altered connections between specific brain areas. Previous studies have investigated ASL-based FC in healthy controls using either ICA-based methods (Jann et al., 2013; Dai et al., 2016) or seed-based analyses (Jann et al., 2013; Storti et al., 2017b, 2018) and reported concordant results with BOLD-based connectivity. However, ASL signals are more directly related to the underlying neuronal activity, being flow-dependent only, and can allow the additional quantification of CBF of specific networks which is not possible by using BOLD only (Jann et al., 2013). Some works have also shown a good spatial similarity between connectivity hubs (from graph-theory metrics) and metabolism/flow distribution, suggesting a close relationship between connectivity and metabolic demands of the brain that can be assessed with ASL data (Liang et al., 2014; Storti et al., 2018). Limited work has been carried out to assess perfusion-based connectivity in patients. As an example, seed-to-voxel perfusion-based FC was assessed in chronic fatigue disease leading to better understanding of this disease’s pathogenesis (Boissoneault et al., 2016).

In epilepsy, while perfusion changes based on ASL CBF maps have already been reported in the epileptogenic area (Wolf et al., 2001; Storti et al., 2014; Boscolo Galazzo et al., 2015, 2016), to the best of our knowledge this is the first work which applied ASL-based connectivity to this neurological condition. Regarding ICA and related RSNs, previous studies on HC have demonstrated the ability of ASL to extract similar RSNs to those identified in BOLD studies, but generally limited to only a subgroup of the most common ones (De Luca et al., 2006). In the most recent study on this topic, Dai et al. (2016) reported seven major group-level RSNs from ASL data in HC, including the DMN, visual networks, SMN, and AUD (Dai et al., 2016), but failed to identify other RSNs as the mesial-limbic structures and THL. Conversely, in our study, we demonstrated for the first time that ASL was able to identify not only the main network (DMN) but also all the others generally detected with BOLD and never previously reported from ASL. These results were confirmed in both controls and patients with 12 RSNs of interest, proving the viability of ASL as an alternative technique for depicting the major RSNs, potentially holding higher specificity/sensitivity than BOLD. While the different networks were qualitatively similar between the two groups, the voxel-wise statistical analysis showed areas of altered connectivity within several ICs. In agreement with previous studies of BOLD FC in epilepsy (Hamandi et al., 2008; Zhang et al., 2009), increased connectivity in the visual areas (occipital and medial visual cortex) was found in PT. Hyperactivity of the visual system and its involvement in seizure propagation in TLE patients have been reported in previous connectivity studies (Hamandi et al., 2008; Zhang et al., 2009). Although this finding is still controversial, this might be interpreted as “hyperfunction” caused by the epileptic activity (Zhang et al., 2009) also in light with the potential connection between epileptogenic areas (parahippocampus and mesial temporal) and the occipital lobe (Hamandi et al., 2008). In our group of patients, areas of increased FC were also shown within networks involved in motor and sensory processes, which have already been proven to be dysfunctional in TLE (Zhang et al., 2009; Cataldi et al., 2013).

Conversely, decreased FC values were found in areas possibly disrupted by the disease, as the DMN and CER. This is in line with previous literature findings, which have shown that the activity in the DMN can be disrupted by the long-term effect of the disease, while decreased connectivity in the cerebellum areas can be related to impaired motor coordination (Zeng et al., 2013). Finally, in the TEMP network (the so-called epileptogenic network in TLE), we found a double pattern, with decreased values ipsilaterally and increased connectivity contralateral to the epileptic focus. This is in agreement with the majority of BOLD rs-fMRI studies that report decreases of connectivity within the epileptogenic network (Centeno and Carmichael, 2014). In addition, Bettus et al. (2009, 2010) showed that in TLE patients FC was also increased in the homologous regions contralateral to the focus, possibly related to the propagation phenomenon. Therefore, the increased connectivity patterns located outside the epileptogenic region in the contralateral hemisphere might suggest a compensatory mechanism in this pathology.

Regarding ROI-to-ROI connectivity, we relied on a functional parcellation of the different nodes based on ICA decomposition and clustering rather than using structural atlases, which have been proven to be only partially suitable for deriving FC matrices (Bijsterbosch et al., 2017). In terms of statistical differences between connectivity matrices, our principal findings are decreased inter-hemispheric FC and increased intra-hemispheric FC (both ipsi- and contra-lateral to the focus) in patients compared to controls. In particular, several within-network connections revealed decreased FC, except for the VIS networks which, however, were characterized by several between-network alterations. Maccotta et al. (2013) reported similar findings, showing decreased local and inter-hemispheric FC together with increased intra-hemispheric connectivity ipsilateral to the focus in unilateral TLE patients. Decreased inter-hemispheric connectivity can be attributed either to adaptation (to preserve function in the contralateral hemisphere) or to disruption (maladaptive organization) as a consequence of the disease (Maccotta et al., 2013). Conversely, increased intra-hemispheric connectivity might represent increased coupling which might be connected to seizure propagation. As expected, the temporal areas showed several dysfunctional links, with the ipsilateral regions revealing increased FC and a decrease in the corresponding right-left coupling.

Although we proved that ASL-based FC can be a feasible tool to study clinical populations and can provide important information in drug-resistant epilepsy, we recognize some limitations. First, the sample size of this study is limited, as we restricted the selection to a group of MRI-negative patients with a clear diagnosis (right TLE). However, in this way, we were able to provide a proof of concept of perfusion-based FC in epilepsy, based on a more homogeneous population. More studies on larger groups, also enclosing other types of epilepsy, are necessary to further evaluate the findings and to better assess the performance of ASL in this pathology. In addition, we lack resting-state BOLD fMRI data which unfortunately were not acquired in all subjects, precluding the possibility to compare our novel ASL-based findings with those coming from a more established FC technique as BOLD. Therefore, in a future study, we will address this important aspect, in order to more precisely evaluate the added value of ASL and compare the information provided by the two imaging modalities.

As additional note of caution, we need to acknowledge that an old ASL sequence was employed in this study (PICORE Q2TIPS ASL product sequence), while the scientific community is moving toward better labeling and readout options (in particular 3D-pCASL) as suggested by the recent consensus paper (Alsop et al., 2015). Moreover, no background suppression pulses were available for our ASL sequence and a rather long TE (17 ms) was chosen which, however, was the shortest possible considering the combination of all the other acquisition parameters and was in line with values previously used in literature. These sequence parameters could include a marked BOLD signal weighting in the ASL images and, combined with the fact the label/control time-series were considered globally rather than subtracted, this could lead to an analysis which is close to a regular BOLD rs-fMRI analysis. Therefore, it is important to keep in mind that our findings might not be directly transferable to more advanced ASL datasets and further studies are necessary to understand the effect of the different sequences and analysis approaches on the connectivity measures derived from ASL.

However, despite its inherent limitations, since the PICORE Q2TIPS ASL product sequence is commercially available in most of the Siemens scanners and is often used in clinical settings where research sequences are rare, we believe it is timely to verify its feasibility and applicability not only for quantifying perfusion but also in the context of connectivity. Our results could add a new piece of information to the current literature, providing some hints about the performance of such scheme for deriving connectivity measures. In particular, we demonstrated how these ASL data can be jointly exploited to derive both connectivity and perfusion estimates. The CBF quantification was left aside for the patient group, as this was out of the main scope of the manuscript, but would provide additional information allowing, among the others, to characterize the CBF levels of the different RSNs and therefore identify the local flow alterations. Finally, regarding the pre-processing steps we decided to focus our analysis on two pipelines only, one of which based on ICA noise removal, as to the best of our knowledge the latter has not been previously applied to ASL data. As future direction, a more complete assessment of the cleaning effect on ASL signals should be performed, including more processing pipelines (as nuisance regression or scrubbing) and, possibly, automated tools for IC classification.

## Conclusion

In conclusion, with this work we aimed at providing a better understanding of ASL pre-processing for removal of noise components as well as to investigating its suitability for the characterization of pathological mechanisms and modulations in epilepsy. Our results proved that ASL-based FC can be a viable technique for characterizing the intrinsic brain organization and its integrity in patients, potentially featuring higher specificity in detecting RSNs than BOLD. The complementary FC analyses provide new insights into the pathological mechanisms characterizing this “network disease” that still remains poorly understood in the current literature. Our findings in terms of dysfunctional areas and connections underline the complex changes occurring in TLE, and stress the need of moving from localization to connectivity as well as to exploit different modalities (such as ASL) for finding a new functional imaging marker for epilepsy.

## Author Contributions

IBG, GM, and FF designed the study, conceived the experiments, interpreted the results, and wrote the manuscript. IBG performed the analyses. SFS and BDB supported the data analysis, interpretation of the results, and drafted the manuscript. AB and EDV were involved in the design of the study, in data acquisition, interpreted the results, and revised the manuscript. MK, JSD, AG, and FBP revised the manuscript and helped in the interpretation.

## Conflict of Interest Statement

The authors declare that the research was conducted in the absence of any commercial or financial relationships that could be construed as a potential conflict of interest.
