# Supplementary material for: Arterial Spin Labeling Reveals Disrupted Brain Networks and Functional Connectivity in Drug-Resistant Temporal Epilepsy
Source: Front Neuroinform. 2019 Mar 6;12:101. doi: 10.3389/fninf.2018.00101 (PMC6414423; doi:10.3389/fninf.2018.00101)
Supplement: Supplementary file 1 [file Table_1.DOCX]

***Supplementary Material***

**Arterial spin labeling reveals disrupted brain networks and functional connectivity in drug-resistant temporal epilepsy**

**Boscolo Galazzo Ilaria, Storti Silvia Francesca, Barnes Anna, De Blasi Bianca, De Vita Enrico, Koepp Matthias, Duncan John, Groves Ashley, Pizzini Francesca Benedetta, Menegaz Gloria and Fraioli Francesco**

*** Correspondence:** Dr. Ilaria Boscolo Galazzo: ilaria.boscologalazzo@univr.it

**2. Supplementary Figures and Tables**

**2.1 Supplementary Tables**

**Supplementary Table 1. Removed components.** The table shows the number of independent components removed by the ICA-based denoising for each HC and run, and their percentage with respect to the total number of components.
